# Hypnotherapy compared to cognitive-behavioral therapy for smoking cessation in a randomized controlled trial

**DOI:** 10.3389/fpsyg.2024.1330362

**Published:** 2024-02-27

**Authors:** Anil Batra, Sandra Eck, Björn Riegel, Sibylle Friedrich, Kristina Fuhr, Iris Torchalla, Sven Tönnies

**Affiliations:** ^1^Department for Psychiatry and Psychotherapy, Section for Addiction Research and Medicine, University Hospital Tuebingen, Tuebingen, Germany; ^2^Private Practitioner, Hohenwestedt, Germany; ^3^Private Practitioner, Quickborn, Germany; ^4^West Coast Resiliency Centre, Vancouver, BC, Canada; ^5^Department of Clinical Psychology and Psychotherapy, University Hamburg, Hamburg, Germany

**Keywords:** smoking cessation, tobacco use, nicotine dependence, cognitive-behavioral therapy, hypnotherapy

## Abstract

Worldwide, more than eight million people die each year as a result of tobacco use. A large proportion of smokers who want to quit are interested in alternative smoking cessation methods, of which hypnotherapy is the most popular. However, the efficacy of hypnotherapy as a tobacco cessation intervention cannot be considered sufficiently proven due to significant methodological limitations in the studies available to date. The aim of the present study was to compare the efficacy of a hypnotherapeutic group program for smoking cessation with that of an established cognitive-behavioral group program in a randomized controlled trial. A total of 360 smokers who were willing to quit were randomly assigned to either hypnotherapy (HT) or cognitive-behavioral therapy (CBT) at two study sites, without regard to treatment preference. They each underwent a 6 weeks smoking cessation course (one 90 min group session per week) and were followed up at regular intervals over a 12 months period. The primary outcome variable was defined as continuous abstinence from smoking according to the Russell standard, verified by a carbon monoxide measurement at three measurement time points. Secondary outcome variables were 7 days point prevalence abstinence during the 12 months follow up and the number of cigarettes the non-quitters smoked per smoking day (smoking intensity). Generalized estimating equations were used to test treatment condition, hypnotic suggestibility, and treatment expectancy as predictors of abstinence. The two interventions did not differ significantly in the proportion of participants who remained continuously abstinent throughout the follow-up period (CBT: 15.6%, HT: 15.0%) and also regarding the 7 days abstinence rates during the 12 months follow-up (CBT: 21.2%, HT: 16.7%). However, when controlling for hypnotic suggestibility, CBT showed significantly higher 7 days abstinence rates. In terms of the continuous abstinence rates, it can be concluded that the efficacy of hypnotherapeutic methods for smoking cessation seem to be comparable to established programs such as CBT.

**Clinical trial registration:**

ClinicalTrials.gov, identifier NCT01129999.

## 1 Introduction

The health consequences of cigarette smoking are well known. Smokers have a higher risk of cancer, cardiovascular disease, and respiratory disease than nonsmokers (GBD 2019 Tobacco Collaborators, 2021). In addition, there is scientific evidence of adverse effects from exposure to secondhand smoke, including cancer and cardiovascular disease in adults and adverse respiratory effects in children and adults. Worldwide, more than eight million people die each year as a result of tobacco use (GBD 2019 Tobacco Collaborators, 2021). Quitting smoking has substantial health benefits for people of all ages and for those with and without smoking-related diseases. It reduces the tobacco-related risks for cancer, heart attack, stroke and chronic lung disease. The health benefits of smoking cessation apply to all stages of the smoking career (Doll et al., 2004). Although the prevalence of tobacco use has decreased over the last decades (Ng et al., 2014; GBD 2019 Tobacco Collaborators, 2021), prevalence rates in Germany are still high, with about 40% of the population identifying as at least occasional smokers (Armstrong, 2023). As awareness of the harmful effects of smoking increases, many smokers report wanting to quit. Thyrian et al. (2008) compared five different countries in terms of smokers’ motivation to quit and found that the majority of smokers expressed motivation to quit (73.5%). The German Study on Tobacco Use (DEBRA) shows a slight downward trend and an overall low level of motivation to quit smoking in Germany with up to 52.4% thinking about quitting but only 24.6% with a desire to quit smoking (Borchardt et al., 2023). It is recommended that smokers who are unable to quit on their own receive professional help (Batra et al., 2022).

Cognitive-behavioral therapy (CBT) is an effective and well-established method for smoking cessation (Batra et al., 2022). CBT is recommended in national treatment guidelines as the treatment of choice in most countries, including Germany (Sucht et al., 2021). Common treatment elements mentioned in this guideline include psychoeducation, self-monitoring, promoting self-efficacy, building social support, and teaching coping and problem-solving skills. Intensive CBT interventions produce acceptable short-term abstinence rates. However, the rates typically decline steadily after the end of therapy, with only about 20% of participants remaining abstinent for one year (e.g., Prochazka, 2000; Rennard and Daughton, 2000; Agboola et al., 2010; McClure et al., 2020). Group therapy interventions were evaluated more effective compared to self-help, and less intensive interventions (Stead et al., 2017). The outcome can be improved with pharmacotherapy (e.g., nicotine replacement therapy), but even with combined strategies, long-term abstinence rates do not exceed 35% (Alterman et al., 2001; Lerman et al., 2004; Batra et al., 2008, 2010; Stead et al., 2016). These results indicate that further research is needed to improve treatment outcomes and identify alternative treatment strategies.

However, some individuals may have strong preferences or disinclination regarding pharmacotherapy or treatment setting and format. It is recommended that treatment preferences should be taken into account when developing a treatment plan, as this has been shown to improve motivation to quit and adherence to treatment (Howard and Thornicroft, 2006; Kleber et al., 2006; Sucht et al., 2021). It might therefore be beneficial to allow smokers to choose from a range of different interventions. In a survey of 1,175 patients at a specialized outpatient tobacco treatment clinic, Sood et al. (2006) assessed smokers’ interest in complementary and alternative medicine for smoking cessation. They found moderate levels of past use (27%) and high interest in future use (67%) of these treatments. Among all respondents, 40% were interested in trying hypnosis to quit smoking. More than 300 current and past smokers (with rheumatoid arthritis) were asked in a survey about their smoking history, their quit attempts and methods they used to quit smoking. Hypnotherapy was listed by them as one of the past complementary or alternative aids (Lopez-Olivo et al., 2023). Tahiri et al. (2012) recommended in their meta-analysis that acupuncture and hypnotherapy should be offered as alternative smoking cessation treatments, especially when conventional aids are refused. Hypnotherapy is already a widely promoted alternative method of smoking cessation. Hypnotherapists assist in changing unwanted behaviors, cognitions, and emotions by inducing hypnotic trance. Hypnotic trance is a state of focused concentration in which individuals are more receptive to suggestions for behavioral change and are able to focus on specific goals. For example, during hypnosis a smoker might receive suggestions to reduce cravings and increase their ability to cope with them (Covino and Bottari, 2001). Imagery plays an important role in visualizing an alternative behavior in the mind (Fromm, 1987). The effects of a trance state on brain activity have been demonstrated in clinical studies (Rainville et al., 2002; Oakley and Halligan, 2009). There is already good evidence for hypnotherapy as a treatment for pain (Elkins et al., 2007) or irritable bowel syndrome (Gonsalkorale et al., 2002).

In hypnotherapy, techniques for smoking cessation, the concept of the “unconscious” together with ideomotor signals can be introduced, to support smokers as a “third party” with identifying the day of quitting smoking or developing ideas to overcome tobacco use. Additional to a theoretical framework that is shared with CBT (e.g., a biopsychosocial model including the concept of a behavior that has an individual, conscious or unconscious function in the smoker’s everyday life, Tidey and Miller, 2015), the “unconscious” can access resources that were perceived as uncontrollable or unavailable (Gerl et al., 2015). Other hypnotic strategies include reframing smoking as conducive (e.g., rewarding, taking a break); using posthypnotic and indirect suggestions and metaphors; using time regression or progression; establishing new rituals such as self-hypnosis; and developing alternatives for potential relapses (Gerl et al., 2015). However, there is still considerable scientific debate about the efficacy of hypnotherapy for smoking cessation. Several randomized trials have compared hypnotherapy with other treatments, such as smoking cessation supported by acupuncture, relaxation, behavioral therapy, or a control condition without an intervention. Valbo and Eide (1996) randomly assigned 158 pregnant women to either hypnotherapeutic treatment or a control condition that received only routine prenatal care. At the time of delivery, the smoking cessation rates for both groups were 10%. In a larger study of 180 participants in a family practice setting, short-term differences in abstinence rates were observed between the hypnotherapy group (21%) and the control group (6%), but no significant differences were found for medium- and long-term abstinence rates (Lambe et al., 1986). The control group in this study received a health booklet on quitting smoking and a medical advice to quit. During follow-up assessments, all patients were called by phone and encouraged while assessing the number of cigarettes smoked. The authors (Lambe et al., 1986) explained the high success rates of the control group in the long term by the personal contact with the interviewers. Some studies have shown hypnotherapy to be more effective than no treatment and as effective as other interventions. For example, Williams and Hall (1988) randomized 60 smokers to either a single session of hypnosis, a placebo control condition (a single session where reasons for quitting and quitting attempts were discussed), or a no-treatment control condition. At posttest and all follow-ups, abstinence rates were significantly higher in the hypnosis group than in either control group. Hyman et al. (1986) randomized 60 smokers to one of four different groups: hypnosis, focused smoking, attention placebo, or a waitlist control group. During focused smoking, participants were instructed to smoke and additionally concentrate on aversive smoking effects. In the attention placebo, participants were discussing their general personal topics. Hypnosis, in this case, consisted of formal trances with specific suggestions mostly related to positive effects of non-smoking. All treatment conditions involved four weekly individual sessions (60 min each). All treatment conditions achieved significantly better abstinence rates than the waitlist control condition (0%), but no significant differences were found between the different interventions. Rabkin et al. (1984) randomized participants to receive either behavior modification (BM), health education (HE), hypnotherapy (HT), or be on the waitlist. Each intervention group was superior to the waitlist control group. There were no significant differences between treatment groups at any follow-up, with abstinence rates of 17% (BM), 19% (HT), and 22% (HE) at the 6 months follow-up. Carmody et al. (2008) randomized 286 participants to receive either standard behavioral counseling or hypnosis, both combined with nicotine patches. Point prevalence abstinence rates did not differ significantly between the two conditions at the 6- and 12 months follow-up. Except the studies of Rabkin et al. (1984) and Carmody et al. (2008), all other previously mentioned RCTs did not involve techniques of CBT in the control groups. Tahiri et al. (2012), stated in their meta-analysis on alternative smoking cessation aids that sample sizes of included studies on hypnotherapy were small, biochemical validation was usually missing, and there were problems with randomization procedures or reporting (Tahiri et al., 2012). In two earlier systematic reviews, the Cochrane Tobacco Addiction Group concluded studies have failed to demonstrate that hypnotherapy produces greater six-month quit rates than other interventions or no intervention, and that the highly significant treatment effects of hypnotherapy on smoking cessation reported in uncontrolled studies could not be confirmed in randomized controlled trials (Abbot et al., 2000; Barnes et al., 2010). Even in the most recent update of the meta-analysis (Barnes et al., 2019) which included 1.926 participants of 14 studies investigating effects of hypnosis compared to various control interventions, the quality of most studies was too low to draw clear conclusions. Of course, it should be noted here that some of the older studies from the 1970s and 1980s used a different definition of hypnotherapy-often using a more direct form of hypnosis than is common today. Techniques have evolved since then and use a resource-based approach. In 2006, the German Scientific Advisory Board for Psychotherapy (Wissenschaftlicher Beirat Psychotherapie [The German Academic Advisory Committee for Psychotherapy], 2006) published a report that included hypnotherapy as an acceptable treatment for tobacco dependence. However, the committee acknowledged that conclusions regarding its efficacy are highly limited due to the heterogeneity of the data. Similarly, the current German guidelines for the screening, diagnosis, and treatment of tobacco abuse and dependence (Sucht et al., 2021; Batra et al., 2022) consider hypnosis as a treatment method that “may be offered” by psychologists or medical doctors with appropriate training, while acknowledging the lack of clarity regarding its indications and contraindications due to limited high-quality evidence. The methodological shortcomings of previous studies include small sample sizes (e.g., Elkins et al., 2006), lack of treatment standardization and manualization, inconsistencies in the definition of treatment outcomes, lack of biochemical validation of abstinence, lack of random assignment to treatment conditions (e.g., Elkins and Rajab, 2004; Riegel, 2013), use of inadequate statistical methods, or failure to report important information regarding the methodology (Abbot et al., 2000; Covino and Bottari, 2001; Barnes et al., 2010, 2019). Others concerns were related to inconsistencies of treatment duration and intensity between the compared treatment conditions (e.g., Wynd, 2005) and lack of active comparison condition (e.g., Elkins and Rajab, 2004). Therefore, researchers have called for higher quality trials of hypnotherapy for tobacco cessation (Flammer and Bongartz, 2003; Sood et al., 2006). The Cochrane Tobacco Addiction Group emphasized that the hypnotherapy intervention used needs to be clearly defined and described, comparison conditions should include active interventions, and the amount of therapist contact time must be matched (Abbot et al., 2000; Barnes et al., 2019).

The aim of the present study was to compare the efficacy of a hypnotherapeutic tobacco cessation program with an established cognitive-behavioral tobacco cessation program. The study was planned in light of the widespread availability of hypnotherapeutic tobacco cessation services and the corresponding high demand for hypnotherapeutic tobacco cessation services – while at the same time, from a scientific point of view, the previous evidence for the effectiveness of the method was found to be insufficient (Abbot et al., 2000; Barnes et al., 2010, 2019). To meet high methodological standards, our study should have the following characteristics: a sufficiently large number of participants, a sufficiently long follow-up period, and a definition of treatment outcome based on the current gold standard of tobacco cessation research, the Russell standard, which includes a biochemical verification of participants’ self-reports (West et al., 2005). The treatment was standardized and manualized, the study project was monitored by an external company, and appropriate statistical methods were used to analyze the data. Since CBT is well evaluated for smoking cessation and more in line with the treatment guidelines than a hypnotherapeutic tobacco cessation treatment which had been adapted in format, setting and duration, we expected that participants receiving CBT would achieve higher abstinence rates than those receiving HT. In addition, selected psychological variables (i.e., therapy expectancy, hypnotic suggestibility) were tested as predictors of study outcome. Therefore, this study should meet the qualitative standards of a randomized controlled trial as required for drug approval.

## 2 Materials and methods

### 2.1 Trial design

In this parallel randomized controlled trial, smokers who were willing to quit and smoked at least 10 cigarettes per day were recruited at two study centers (Tuebingen and Hamburg). Eligible participants were randomly assigned 1:1 in blocks of eighteen to receive either six weeks of cognitive behavioral therapy (CBT) or six weeks of hypnotherapy (HT). Both interventions were outpatient, group-based (with eight to nine participants per group), and were delivered in weekly 90 min sessions with a trained therapist. After completion of treatment, participants were reassessed 1, 3, 6, 9, and 12 months following treatment completion. Participants received 10 euros as compensation for their in-person participation at the 1 month and 12 months follow-up, and they received additional 50 euros at the 12 months follow-up if they had completed all follow-up assessments. The study was conducted in accordance with Good Clinical Practice (GCP) guidelines and the Declaration of Helsinki. Ethical approval for this project was obtained from the Ethics Committee for Behavioral Research of the Medical Faculty of the Eberhard Karls University of Tuebingen (331/2008B01) and the Ethics Committee of the Medical Association of Hamburg (MC-150/10). The study has been pre-registered at ClinicalTrials.gov (NCT 01129999).

### 2.2 Trial sample

Criteria for eligibility included being at least 18 years old, smoking at least 10 cigarettes per day for at least the past 2 years, being able to communicate and be understood in German, and being able and willing to provide written informed consent. Exclusion criteria were a serious mental disorder (i.e., life-time psychotic disorder, bipolar disorder, posttraumatic stress disorder, dissociative disorder, current episode of major depression, current alcohol or drug dependence, borderline personality disorder), use of tobacco products other than cigarettes (cigarillos, cigar, pipe), participation in any smoking cessation treatment within the past 6 months, and current status of pregnancy or lactation. Participants with severe mental disorders were excluded because they might need additional pharmacological treatment or support for quitting smoking (e.g., El-Guebaly et al., 2002) and differ from individuals without a history of mental disorders by lower success rates (Peckham et al., 2017). Furthermore, some mental disorders, as for example psychotic disorders, are listed as a contraindication for hypnotherapy (e.g., Walker, 2016).

**Figure 1:**
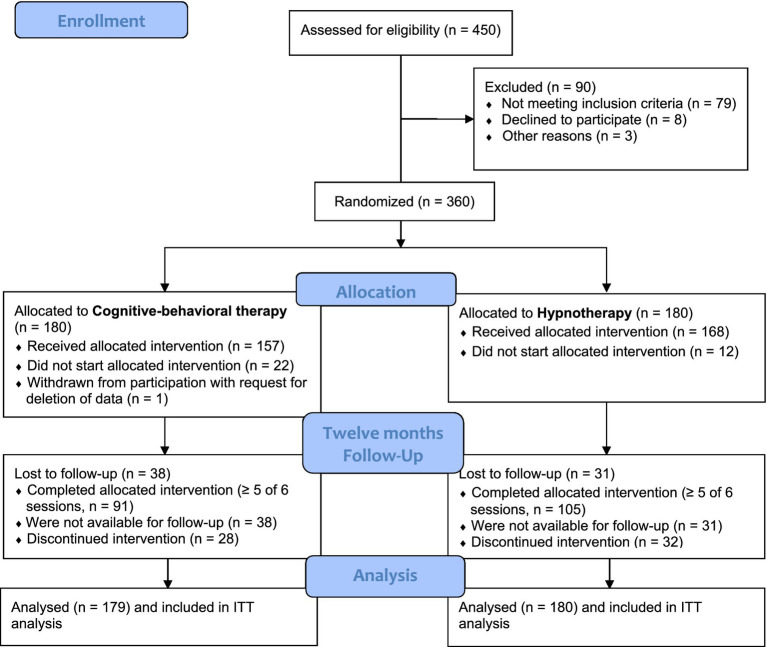
CONSORT diagram. Trial enrollment, randomization, and follow-up.

Between September 2010 and January 2012, a total of 450 adult smokers were screened for inclusion and exclusion criteria at both study sites. Of those interested in participating in the study, 371 (82.4%) were eligible based on these criteria. Of those who were eligible, a small number later refused to participate (*n* = 8) or could not be contacted (*n* = 3). The remaining 360 participants were randomly assigned to receive either the CBT or HT smoking cessation program (CBT: *n* = 180, HT: *n* = 180, see also Figure 1). One participant withdrew from the study and requested his data to be deleted, so the intention-to-treat (ITT) sample consisted of *N* = 359 participants (CBT: *n* = 179). Once randomized, there was no deviation from the assigned treatment condition. Figure 1 shows the CONSORT diagram.

Table 1 describes the characteristics of the total sample and the treatment conditions. Participants (59.9% female) were on average 43.07 years old (SD = 12.52). They started smoking regularly at a mean age of 16.53 years (SD = 3.00). At baseline, they smoked an average of one pack of cigarettes per day (number of cigarettes: M = 19.75, SD = 6.86). The two treatment conditions did not differ significantly at baseline in any of the baseline variables measured. The general efficacy expectation for HT treatment was rated significantly higher than the efficacy expectation for CBT treatment before randomization (*z* = −0.595, *p* < 0.001). Preferences of participants for treatments were also assessed at baseline in 249 participants (another 110 did not answer this question). Of these, most participants preferred HT (*n* = 175, 70.3%) over CBT (*n* = 31, 12.4%), some had no preference (*n* = 43, 17.3%).

**Table 1:** Sample characteristics and comparison of treatment conditions.

	Total (*N* = 359)	CBT (*n* = 179)	HT (*n* = 180)
Sex assigned at birth, *n* (%) women	215 (59.9)	101 (56.4)	114 (63.3)
Age (in years)	43.07 (12.52)	43.28 (12.18)	42.86 (12.88)
Smoking intensity (cigarettes/day)	19.75 (6.86)	20.35 (6.85)	19.15 (6.83)
Smoking duration (in years)	26.54 (12.36)	26.76 (12.17)	26.32 (12.57)
Age at the start of smoking (in years)	16.53 (3.00)	16.53 (2.98)	16.54 (3.04)
Level of nicotine dependence (FTND, *N* = 317)	6.02 (1.71)	6.13 (1.70)	5.91 (1.72)
“How important is it to you to become smoke-free at the moment?” (MQ)	8.54 (1.57)	8.45 (1.68)	8.62 (1.45)
“How confident are you that you can achieve this?” (MQ)	6.12 (1.98)	5.93 (2.09)	6.29 (1.86)
“How high would you rate the general efficacy of CBT?” (STEQ)	6.28 (1.77)	6.28 (1.79)	6.28 (1.75)
“How high would you rate the general efficacy of HT?” (STEQ)	6.88 (1.91)	6.74 (1.92)	7.03 (1.90)
Hypnotic suggestibility (HGSHSA)	6.68 (2.47)	6.53 (2.54)	6.83 (2.39)

	Total (*N* = 249)	CBT (*n* = 140)	HT (*n* = 109)
	*n* (%)	*n* (%)	*n* (%)
Previous smoking cessation attempts, *n* (%) yes	318 (88.6)	160 (89.4)	158 (87.8)
Preference for HT	175	97	78
Preference for CBT	31	19	12
No preference	43	24	19

Mean values and standard deviations are shown unless otherwise stated. FTND, Fagerström test for Nicotine dependence; MQ, motivation questionnaire, answered on a 10-point Likert scale (1 = not at all, 10 = very); STEQ, the subjective treatment efficacy questionnaire (1 = not at all, 10 = very); HGSHS-A, Harvard group scale of hypnotic susceptibility – Form A. All n.s.

### 2.3 Assessments

#### 2.3.1 Baseline assessments

At baseline, sociodemographic variables, smoking behaviors, hypnotic susceptibility, motivation to quit smoking, and expectations for treatment with CBT and HT were assessed. The following instruments were used:

The Fagerström Test for Nicotine Dependence (FTND; Heatherton et al., 1991; Schumann et al., 2003), a 6-item self-report questionnaire, was used to assess participants’ baseline level of nicotine dependence. Responses are summed to produce a total score between 0 and 10. Previous studies have established the reliability and validity of this measure in English and German speaking samples (Heatherton et al., 1991; Schumann et al., 2003).

The Harvard Group Scale of Hypnotic Susceptibility (HGSHS) Form A (Shor and Orne, 1963) was used to assess hypnotic susceptibility in groups. Participants listened to a standardized audiotape recording by Bongartz (1985) that starts with a relaxation induction followed by instructions for eleven tasks, e.g., an immobilization of the right arm: suggestion included imagination of heaviness spreading all over the body with focus on the right arm until the hand is too heavy to move, even when the subjects wants to lift the hand. The participant then rated the performance of the tasks on a binary scale. The HGSHS score ranges from 0 to 11. German norms have been evaluated by Bongartz (1985).

Abstinence motivation was assessed with two items measuring the perceived importance of quitting smoking and participants’ confidence in their ability to achieve/maintain abstinence on a 10-point Likert scale. The items were based on suggestions by Miller and Rollnick (2002). Participants were asked: “How important is it to you to become smoke-free right now?” (1 = not at all, 10 = very) and “If you were to decide to become smoke-free now, how confident are you that you can achieve this?” (1 = not at all, 10 = absolutely). These items have been used in previous studies to assess motivation to quit smoking (Batra et al., 2008, 2010) and were named Motivation Questionnaire (MQ).

Overall efficacy estimation of CBT and HT was assessed at baseline using two items that were answered on a 10-point Likert scale (1 = not at all, 10 = very). These Subjective Treatment Efficacy Questions (STEQ) were asked at baseline (“How high would you rate the general efficacy of CBT” and “How high would you rate the general efficacy of HT?”).

#### 2.3.2 Follow-up assessments

The Follow-up Smoking Questionnaire (Batra and Buchkremer, 2011) was used to assess self-reported smoking status at each follow-up time point: 1, 3, 6, 9, and 12 months post-treatment. Seven-day point-prevalence abstinence (PPA) and continuous abstinence based on the Russell Standard (West et al., 2005) were assessed via self-report. For current smokers, further details of their smoking behaviors were explored. Self-reported smoking status was validated by exhaled carbon monoxide (CO) measurements using the piCO smokerlyzer (Bedfont, England) at the end of treatment and at the 1- and 12 months follow-up visits. In accordance with the Russell standard (West et al., 2005), a CO measurement of 10 or more parts per million was defined as indicative of current smoking. Follow-ups were conducted by study assistants who were blinded to participants’ study condition.

#### 2.3.3 Primary outcome measure

The primary outcome measure was 12 months continuous abstinence (CA) according to the Russell standard (West et al., 2005). This is defined as self-report of having smoked no more than five cigarettes since the end of treatment (and during all assessments 1, 6, 9, and 12 months later), supported by negative biochemical validation (CO < 10 ppm).

#### 2.3.4 Secondary outcome measures

Secondary outcome measures were self-reported 7 days point-prevalence abstinence (PPA) 1, 6, 9, and 12 months after the end of treatment.

We also asked non-quitting smokers about the number of cigarettes they smoked per smoking day (smoking intensity).

In addition, treatment compliance was assessed via session attendance. Treatment compliance was defined as attending at least five out of six scheduled treatment sessions, consistent with previous work (Batra et al., 2008, 2010). Attendance at follow-ups was also assessed.

Safety-critical events were defined as: suicidal thoughts/wishes, or moderate or strong feelings of sadness or depression, both assessed in the questionnaires at the visits and follow-ups; serious mood reduction or new psychiatric symptoms, both assessed during the treatment phase by one of the therapists. In case of a safety-critical event, the event was documented by the therapists or study assistants for the follow-up assessments, faxed to the PI of the study, and discussed at team meetings.

### 2.4 Interventions

Both treatment programs were matched in terms of contact time and therapy format and were delivered in six weekly group sessions of 90 min each, with seven to nine participants per group. The 6 weeks were chosen, because the standard CBT program used in the RCT has a duration of 6 weeks. Both treatments were manualized. Interventions were delivered by master’s level clinical psychologists who had received additional training in the cognitive-behavioral or the hypnotherapeutic smoking cessation treatment manual. Both treatment programs began with a preparatory phase while participants were still smoking (sessions 1 and 2). In both conditions, smokers were encouraged to set a quit date at any time between sessions 2 and 3. Sessions 3–6 provided support for maintaining abstinence. The content of each session differed according to the underlying rationale for the intervention. Between October 2010 and February 2012, a total of 40 smoking cessation groups were held at both study sites (20 groups per side, 10 receiving CBT, 10 receiving HT). The content of each session for both programs are detailed in the Supplementary material. No pharmacological support was offered for either intervention.

#### 2.4.1 Cognitive-behavioral therapy

The CBT Smoking Cessation Group Program was developed by the Smoking Cessation Research Group at the Department of Psychiatry and Psychotherapy, University of Tübingen, Germany, and has been evaluated in a number of studies (Batra et al., 1994; Schröter et al., 2006; Batra et al., 2008, 2010). It has been published both as a smoking cessation manual for therapists (Batra and Buchkremer, 2004) and as a self-help manual for smokers (Batra and Buchkremer, 2006/2008). It is approved by the German Medical Association as an effective smoking cessation program.

The program includes the following components: psychoeducation, self-monitoring of smoking behavior, identification of smoking cues and smoking-related situations, functional analysis of smoking behavior, motivational enhancement strategies (e.g., weighing the pros and cons of smoking and quitting), developing alternative behavioral options, self-control and stimulus control strategies, reinforcement of abstinence, strategies for coping with smoking urges and withdrawal symptoms, social support/social contracts, strategies for preventing weight gain, encouragement of physical activity, relaxation, relapse prevention strategies, and relapse management strategies.

#### 2.4.2 Hypnotherapy

The hypnotherapy program is based on two standardized smoking cessation manuals (Gerl, 1997; Schweizer, 2009; last updated: Gerl et al., 2015). The program includes the following components: trance-induced focusing on the desired internal and external state, development of a positive self-perception (smokers are supported to create a sense of a positive future without cigarettes), reframing of smoking behaviors and relapses, finding a suitable quitting date using ideomotor actions, self-empowering suggestions and metaphors, development of new rituals, posthypnotic suggestions to connect the cognitive and emotional experiences of trance with daily life, and self-hypnosis to imagine life without cigarettes.

### 2.5 Procedure

Participants were recruited through advertisements in local media, flyers mailed to primary care providers, and university-wide email campaigns between September 2010 and January 2012. Individuals interested in participating were mailed a detailed information sheet and invited to attend an information session at the local study center. During the information session, they were provided with details about the goals and rationale of the study, the requirements for participation, and their rights as participants. They also had the opportunity to ask questions about their participation in the study. They were then asked to provide written informed consent. Subsequently, inclusion and exclusion criteria were reviewed to assess eligibility for the study. Study participants received a study code number and completed baseline questionnaires (FTND, MQ, STEQ, and the HGSHS hypnotic suggestibility test). Non-eligible individuals were offered participation in a regular smoking cessation program outside of the study. Study participants were randomly assigned to receive either CBT or HT for smoking cessation, regardless of treatment preference or hypnotic suggestibility. Participants were randomized simultaneously 1:1 to either CBT or HT in blocks of eighteen subjects. Two groups of participants were formed, 50% of whom were assigned to treatment option 1 (CBT) and 50% to treatment option 2 (HT). Randomization was performed with nQuery 2.0 (Statsols, Cork, Ireland) by the Institute for Clinical Epidemiology and Biometry (ICEAB), Tübingen. Allocation was concealed until completion of the baseline assessments. Both study centers were informed of the outcome of the randomization process by fax. Participants were informed of the outcome of the randomization process by telephone and were invited to attend a specific smoking cessation course. Possible course dates were discussed with each participant prior to randomization, i.e., participants were randomized only if they confirmed the scheduled course dates. At the end of the 6 weeks active treatment period, participants were followed for 12 months. In-person follow-up assessments were conducted at the 1 month and 12 months follow-up assessments by five study assistants, which were psychologists with a diploma or master’s degree. They were blind regarding treatment allocation. Questionnaires were mailed to participants for the 3-, 6-, and 9 months reassessment, with a request to return the assessment forms within 14 days. Participants who did not return the questionnaires on time were contacted and interviewed by telephone. If no information could be obtained within 4 weeks, participants were coded as non-abstinent at that follow-up time point.

The ICEAB also provided an internet-based data entry platform, which was used for this study. All data were collected by paper and pencil and entered twice by two independent study assistants (five in total, all students of psychology) who had received extensive training from the ICEAB.

The study was monitored by an independent company (CENTRIAL GmbH). Monitoring included controlling the patient identity lists and informed consent documents, reviewing inclusion and exclusion criteria of every second patient, supervising the randomization procedure, ensuring proper documentation and compliance with the study protocol, reviewing deviations from the study protocol if any (for example taking pharmacological treatment during nicotine withdrawal), and monitoring safety-critical events. In total, there were three planned visits per center and year plus an initial visit before study start.

### 2.6 Statistical analysis

#### 2.6.1 Power analysis

Sample size calculation was based on the results of previous studies (e.g., Batra et al., 2008), in which the marginal probabilities for abstinence with CBT were *p* = 0.60. Given the lack of reliable data for HT, a medium effect size was assumed. For the sample size determination, it was assumed that continuous abstinence in the group of participants with HT will be 15% and that the case number should be sufficient to detect a clinically significant difference to CBT with a power of 80% and a significance level of 5% (two-sided). Using these assumptions, the number of cases per treatment condition would be *n* = 121 (Calculated with nQuery 4.0, panel PTT0-1, Statsols, Cork, Ireland). To account for cluster structure, an inflation factor must be considered, which is derived from assumptions about inter-cluster correlation (*κ*), as well as the correlation of outcomes within a cluster, and the size of the clusters. The inflation factor is calculated as IF = 1 + (*m* − 1) *κ*, where *m* is the number of individuals per cluster (Donner et al., 1981). This formula shows that even small correlations within the cluster have a large impact on the number of cases. On the other hand, there are no empirical data on the inter-cluster correlation within corresponding clusters formed by group therapy. There is evidence that there is little relationship between individuals in a randomly assembled group therapy cluster with respect to the relevant therapeutic outcome. We therefore made an assumption of *κ* = 0.05. With a constant cluster size of *n* = 8, this results in an inflation factor of 1.35 and thus a corrected case number of *n* = 164 per therapy group. This corresponds to 21 group therapy clusters (*n* = 168) per therapy arm, since not all of them have to be completely filled with eight participants for the evaluation. Therefore, a case number of 336 participants is required. According to the Russel standard, only deceased participants or participants of whom it is unknown whether they are still alive are considered drop-outs. It can therefore be assumed that the number of 21 group therapy clusters with a planned number of 8 participants each is sufficient and accounts for potential drop-outs.

#### 2.6.2 Statistical analysis

For the primary and secondary outcomes, all randomized ITT participants (*N* = 359) were analyzed. Study participants who (a) did not attend a follow-up visit, or (b) did not have a CO measurement, or (c) whose measured CO value exceeded the threshold of 9 ppm defined as critical (West et al., 2005) were classified as smokers. Continuous abstinence and 7 days point prevalence abstinence (PPA) were coded as 0 = not abstinent, 1 = abstinent. For the primary and secondary outcomes, the number and percentage of participants who were abstinent will be reported.

The primary analysis with confirmatory objective was performed using a population-averaged generalized estimating equation (GEE; Liang and Zeger, 1986) model to predict continuous abstinence at the 12 months follow-up. The cluster effect was considered by adjusting for the factor “group-therapeutic cluster.” This procedure was primarily used to estimate the effect of treatment condition (HT vs. CBT) and to examine the null hypothesis that both therapy methods are equally effective. An intervention is considered superior to the other if the *p*-value of the test is smaller than the predefined significance level of 5%.

To test whether continuous abstinence at the 12 months follow-up interview was predicted by time (including all timepoints), hypnotic suggestibility, and treatment condition, population-averaged GEE models were constructed and tested using STATA (version 10). A linear time variable (*t*), a squared time variable (*t*^2^), suggestibility, and treatment condition (0 = HT, 1 = CBT) were included in the models as predictor variables. Continuous abstinence (CA) and 7 days point prevalence abstinence (PPA) were used as criterion variables (each coded 0 = not abstinent, 1 = abstinent). Due to the dichotomous nature of the criterion variables, a Bernoulli distribution was used as the basis and the logit link function was selected, and a variable correlation structure was established due to multiple, unequally distributed survey time points. In addition, models were built that included the interaction terms in addition to the main variables. Two sets of linear predictors were fit to the data (models including treatment condition and models adding time × treatment condition interactions) to search for differential treatment effects over time while accounting for hypnotic susceptibility. The quasi-likelihood under independence model information criterion (QICu) was used to determine which of the models best fit the data (Hardin and Hilbe, 2003). GEE models were constructed and tested again that included first hypnotic suggestibility and second therapy expectancy as additional predictors. Missing data in the GEE models were due to missing data in the suggestibility (HGSHS-A) or therapy expectancy at baseline.

Treatment compliance, attendance at follow-up, the 7 days PPA and CA at the end of the treatment and at the 6 months follow-up as well as safety-critical events are reported as numbers and percentages for all participants.

## 3 Results

### 3.1 Treatment compliance and safety

On average, one in nine participants did not attend a single treatment session (see Figure 1). All others, *n* = 325 (90.5%), entered the treatment to which they were randomly assigned and attended at least one of the six scheduled treatment sessions (*n* = 157 (87.7%) in the CBT condition and *n* = 168 (93.3%) in the HT condition). In total 54.6% of the sample was compliant according to this definition; 50.8% (*n* = 91) of the CBT condition and 58.3% (*n* = 105) of the HT condition. The difference between the two treatment conditions was not statistically significant, *p* > 0.05.

In total, 34 safety-critical events were assessed, documented, and monitored. These included 30 reports of feeling of sad or depressed, 15 in HT and 15 in CBT, and two events involving suicidal thoughts. Two events were reported by therapists during treatment, with one person reporting serious mood reduction and one person with new psychiatric symptoms (depressive episode of a bipolar disorder). All cases were discussed with the PI of the study, but it was not necessary for the study team to take any action.

### 3.2 Attendance at follow-up visits

**Figure 2:**
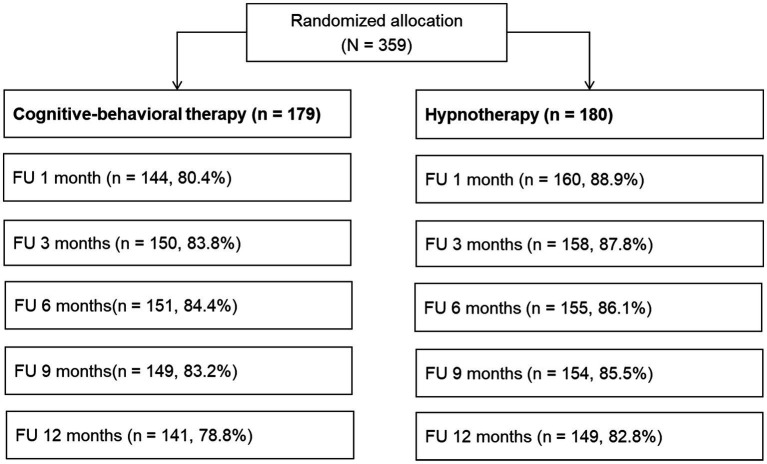
Participation and attendance rates at follow-ups.

In total 84.7, 85.8, 85.2, 84.4, and 80.8% of study participants could be reached for the follow-up visits 1, 3, 6, 9, and 12 months after treatment completion. Attendance rates by treatment condition are shown in Figure 2. Attendance rates differed significantly between treatment conditions at the 1 month post-treatment follow-up (FU1; *χ*^2^(1) = 4.930, *p* < 0.05). Fewer subjects in CBT (80.4%) attended FU1 compared to HT (88.9%). Attendance rates were not significantly different at all other follow-up visits (all *p* > 0.05). The on-site appointment to measure the carbon monoxide level in the breath was completed by 66.6 71.0% at FU1 and FU12, respectively without significant differences between treatment conditions (all *p* > 0.05).

### 3.3 Continuous and point prevalence abstinence rates during the follow-up phase

**Figure 3:**
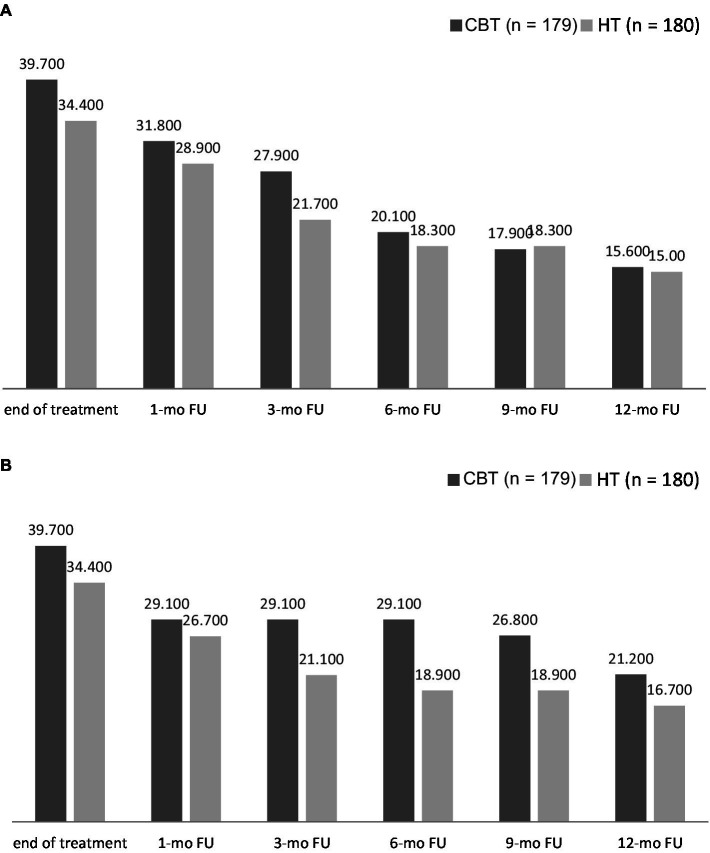
**(A)** Continuous abstinence according to Russell standard over the course of the 12 months follow-up period. **(B)** Seven-day point-prevalence abstinence over the 12 months follow-ups period.

At the end of treatment 39.7% of participants in the CBT condition and 34.4% of participants in the HT condition were classified as abstinent. Figures 3A,B show the CA rates and the 7 days PPA rates at all follow-up visits during the 12 months follow-up period.

Self-reported CA validated with CO measurement 6 months after treatment completion were 20.1% in the CBT condition and 18.3% in the HT condition, and 15.6% (*n* = 28) in the CBT condition and 15.0% (*n* = 27) in the HT condition at the 12 months follow up assessment.

The 7 days PPA 6 months after treatment completion was 29.1% (CBT) and 18.9% (HT). The 7 days PPA validated with CO measurement at the 12 months follow up assessment were 21.2% (CBT) and 16.7% (HT), respectively.

In the whole sample, abstinence rates were also higher as a function of the number of therapy sessions attended. For example, the 7 days PPA at the end of treatment was 15.8% for participants attending four therapy sessions, 40.7% for those attending five sessions, and 48.2% for those attending all six sessions. At the 12 months follow-up, the 7 days PPA was 11.8, 17.4, and 37.3% for participants attending four, five, and six sessions, respectively. Adding the predictor therapy adherence (number of sessions attended) to a logistic regression with the 12 months 7 days PPA as outcome and the predictor therapy condition revealed a significant effect (*χ*^2^(1) = 41.473; *p* < 0.001).

### 3.4 Therapy condition as predictor of smoking abstinence

For the primary analysis, that was an effect of therapy condition on CA over time, the main effects model described the data best, (*χ*^2^(6, *N* = 359) = 56.40, *p* < 0.001; QIC = 482.41), with time (*t*) and squared time (*t*^2^), but not therapy condition (OR = 0.73, *p* = 0.126, 95% CI = 0.49–1.09) as significant predictors (see Table 2). GEE models with the additional predictors sex and age did not show any significant contribution to the results.

**Table 2:** Prediction of treatment abstinence rates after 12 months, ITT sample (*N* = 359).

	Odds ratio	Std. Err	*z*	*p*	95% CI
Continuous abstinence according to Russell standard					
*t*	0.847	0.026	−5.46	<0.001	0.798–0.899
*t* ^2^	1.007	0.002	3.67	<0.001	1.003–1.011
Treatment condition	0.733	0.149	−1.53	0.126	0.493–1.091
Seven-day point prevalence abstinence					
*t*	0.915	0.028	−2.89	0.004	0.861–0.972
*t* ^2^	1.003	0.002	1.27	0.204	0.998–1.007
Treatment condition	0.760	0.146	−1.43	0.152	0.521–1.107

*t*, time variable; *t*^2^, squared time variable; Treatment condition (0 = HT, 1 = CBT); CI, confidence interval.

When 7 days PPA was used as the criterion variable, the main effects model also described the data better than the interaction model (*χ*^2^(6, *N* = 359) = 37.42, *p* < 0.001; QIC = 414.84) with the significant predictor time (*t*), but neither squared time (*t*^2^), nor therapy condition (OR = 0.76, *p* = 0.152, 95% CI = 0.52–1.11) were significant predictors, see Table 2.

### 3.5 Hypnotic suggestibility as additional predictor of smoking abstinence

When CA was used as the criterion variable with therapy condition and hypnotic suggestibility, again the main effects model described the data better than the interaction model, *χ*^2^(6, *N* = 310) = 73.63, *p* < 0.001; QIC = 230.05, with time (*t*) and squared time (*t*^2^) emerging as significant predictors. GEE analyses showed no significant effect of treatment condition (OR = 0.66, *p* = 0.053, 95% CI = 0.43–1.00) or suggestibility (OR = 1.01, *p* = 0.821, 95% CI = 0.93–1.10) on CA over time, see Table 3.

**Table 3:** Prediction of treatment abstinence rates after 12 months with predictors suggestibility and treatment condition, total sample (*N* = 310).

	Odds ratio	Std. Err	*z*	*p*	95% CI
Continuous abstinence according to Russell standard					
*t*	0.856	0.027	−4.95	<0.001	0.805–0.910
*t* ^2^	1.006	0.002	2.98	0.003	1.002–1.010
Treatment condition	0.661	0.141	−1.94	0.053	0.435–1.005
Suggestibility	1.010	0.043	0.23	0.821	0.928–1.098
Seven-day point prevalence abstinence					
*t*	0.910	0.030	−2.84	0.004	0.853–0.971
*t* ^2^	1.003	0.002	1.39	0.164	0.999–1.008
Treatment condition	0.661	0.135	−2.02	0.043	0.443–0.987
Suggestibility	1.017	0.041	0.43	0.668	0.941–1.100

*t*, time variable; *t*^2^, squared time variable; suggestibility (centered); treatment condition (0 = HT, 1 = CBT); CI, confidence interval.

Using the additional predictor hypnotic suggestibility, the effect of the main model was significant, *χ*^2^(6, *N* = 309) = 34.01, *p* < 0.001; QIC = 323.66. Time (*t*), but not squared time (*t*^2^), proved to be a significant predictor of PPA over time, and suggestibility again showed no significant effect. In contrast, treatment condition (controlling for the influence of time and suggestibility) emerged as a significant predictor of PPA: CBT participants had a 6.6% increased chance of abstinence compared to HT (OR = 0.66, *p* = 0.043, 95% CI = 0.94–1.10, see Table 3).

### 3.6 Therapy expectancy as additional predictor of smoking abstinence

The same procedure was used to test therapy expectancy as a predictor of abstinence. This was asked prior to treatment in the form of a subjective assessment of the general effectiveness of each treatment method. The results of the model test are presented in Table 4. Subjective assessment of the overall effectiveness of CBT before treatment began was a significant predictor of abstinence during treatment (OR = 1.27, 95% CI = 1.11–1.45), as was assessment of the effectiveness of HT, but in the opposite direction (OR = 0.87, 95% CI = 0.78–0.98). They show that a higher expectation of the effectiveness of CBT was associated with a higher overall probability of abstinence, whereas a higher estimate of the effectiveness of HT was associated with a lower probability of abstinence. This finding was independent of the definition of abstinence used (see Table 4).

**Table 4:** Prediction of treatment abstinence rates after 12 months with predictors suggestibility, treatment condition, efficacy of CBT and HT, total sample (*n* = 256).

	Odds ratio	Std. Err	*z*	*p*	95% CI
Continuous abstinence according to Russell standard					
*zt*	0.892	0.013	−7.93	<0.001	0.867–0.918
*zt* ^2^	1.001	0.002	3.77	<0.001	1.004–1.013
Suggestibility	1.018	0.045	0.42	0.677	0.935–1.110
Treatment condition	1.376	0.297	1.48	0.139	0.902–2.101
Efficacy expectation CBT	1.270	0.086	3.51	<0.001	1.111–1.451
Efficacy expectation HT	0.872	0.051	−2.34	0.019	0.779–0.978
Seven day point prevalence abstinence					
*zt*	0.931	0.013	−5.17	<0.001	0.906–0.957
*zt* ^2^	1.004	0.003	1.58	0.114	0.999–1.009
Suggestibility	1.013	0.042	0.31	0.760	0.934–1.098
Treatment condition	1.471	0.307	1.85	0.064	0.977–2.214
Efficacy expectation CBT	1.263	0.079	3.73	<0.001	1.117–1.427
Efficacy expectation HT	0.883	0.048	−2.30	0.021	0.794–0.982

*zt*, centered time variable; *zt*^2^, squared centered time variable; suggestibility (centered); treatment condition (0 = HT, 1 = CBT); HT, hypnotherapy; CBT, cognitive-behavioral therapy; CI, confidence interval; efficacy expectation: “How high would you rate the general efficacy of CBT/HT?”

### 3.7 Smoking intensity over the 12-months follow-ups period

As another secondary outcome measure, we also compared the smoking intensity (cigarettes smoked per smoking day in non-quitters) between CBT and HT during the follow-up period (see Table 5). The number of cigarettes per day was reduced compared to baseline especially at the 1 month follow-up. Afterwards on average around 14 cigarettes were smoked in both conditions, CBT and HT, resulting in a reduction of 5–6 cigarettes compared to baseline, thus indicating a reduced harm for participants in the study who continued smoking. There were no differences between the therapy conditions (all n.s.).

**Table 5:** Self-reported smoking intensity (cigarettes/day) over the 12 months follow-up periods in non-quitters.

	Smoking intensity (cigarettes/day)		
	Total	CBT	HT
Baseline	19.75 (6.86) (*N* = 359)	20.35 (6.85) (*n* = 179)	19.15 (6.83) (*n* = 180)
1 mo FU	12.19 (7.76) (*N* = 181)	12.31 (8.21) (*n* = 80)	12.10 (7.42) (*n* = 101)
3 mo FU	13.87 (7.41) (*N* = 207)	14.14 (7.44) (*n* = 94)	13.65 (7.40) (*n* = 113)
6 mo FU	14.51 (7.26) (*N* = 221)	14.79 (6.57) (*n* = 102)	14.27 (7.82) (*n* = 119)
9 mo FU	14.71 (7.28) (*N* = 223)	14.71 (7.25) (*n* = 106)	14.72 (7.34) (*n* = 117)
12 mo FU	13.98 (7.07) (*N* = 219)	14.41 (7.44) (*n* = 102)	13.60 (6.74) (*n* = 117)

Mean values and standard deviations are shown unless otherwise stated. Participants were asked how many cigarettes they smoked per “smoking day.” All *t*-tests were n.s.

## 4 Discussion

To the best of our knowledge, the study presented here can substantially contribute to and enhance the literature on the efficacy of hypnotherapeutic tobacco cessation treatment since it comprises a randomized controlled trial comparing hypnotherapy with an established cognitive-behavioral group therapy, meeting high methodological standards in a large sample.

The primary outcome variable was continuous abstinence according to the Russell standard 12 months after the end of treatment, confirmed by an objective measure, the CO concentration in the exhaled air. Continuous abstinence rates were similar in the CBT condition and the HT condition. Regarding the primary outcome, our main hypothesis assuming superiority of CBT over HT was not confirmed. For the secondary outcome 7 days PPA, CBT was superior to HT but only in the GEE model when controlling for time and hypnotic suggestibility. The results, thus, seem to indicate that there is overall no difference in the effectiveness of the two treatment conditions in achieving and maintaining continuous abstinence from tobacco after RS and also in the 7 days PPA and number of cigarettes smoked by non-quitters (secondary endpoints). Comparisons with the results of previous studies on the efficacy of HT are limited because many of the previous studies had considerable methodological flaws (Barnes et al., 2010, 2019). In the RCT by Carmody et al. (2008), the PPA 12 months after treatment completion was 20% in the hypnosis condition and 14% in the behavioral counseling condition, but the differences were not statistically significant. At first glance, this contradicts the finding of the present study. However, the treatment intensity in the present study, with six treatment sessions of 90 min each, was significantly higher than in the study by Carmody et al. (2008) with only two sessions. Additionally, in the study by Carmody et al. (2008) both intervention conditions were combined with the use of a nicotine patch, whereas in the present study no pharmacotherapeutical support was used. Since the present study had been started, additional RCTs on hypnosis for smoking cessation have been initiated. Searching the international and national clinical registers, two studies were found in clinicaltrials.gov that were comparable to the present study. For example, Carmody et al. (2017) conducted another RCT comparing two sessions of hypnosis with behavioral counseling in 102 smokers (NCT00770380). They found no statistical differences in the 7 days PPA between hypnosis (42%) and the behavioral counseling group (43%) after 12 months, 29 and 28%, respectively, after biochemical validation using saliva (Carmody et al., 2017). Another study (NCT04899492) was registered where recruiting was still “ongoing” although completion date was planned September 2023. In this study, a total of 100 patients with different types of cancer willing to quit smoking before surgery will be randomized to either Motivational Interviewing with CBT, Motivational Interviewing with HT, or to the control group, a nicotine replacement therapy. Outcome will be the 7 days PPA confirmed by a CO measurement. Results are not yet available. In a pilot study with 30 participants, who were randomized to either hypnosis or a nicotine replacement therapy, a trend was reported to suggest that hypnosis was more effective in reducing the number of cigarettes (Lourmière et al., 2022). A recent COCHRANE umbrella review included previous reviews on smoking cessation, but still concluded uncertainty about the effects of hypnosis (Hartmann-Boyce et al., 2021).

The results of the present study, even though not published at that time, were also included in the review of Barnes et al. (2019). Barnes et al. (2019) included the results of this study in the analysis where hypnotherapy was compared to “attention-matched” behavioral interventions. No differences were found at follow-ups between HT and the active control groups regarding abstinence rates. Our results are, thus, in line with those of other studies included in the COCHRANE review (Barnes et al., 2019). Based on our own study results, we conclude that HT was not inferior to CBT which is considered the “gold-standard” treatment for smoking cessation.

The potential superiority of CBT over HT with regard to PPA (even though only found when controlling for hypnotic suggestibility) may be attributed to the relapse management strategies which were included in the CBT-program but not at this intensity in the HT program, designed to support individuals to return to tobacco abstinence after a setback or relapse. Future studies will have to show whether the superiority of CBT over HT with regard to PPA, as found in the present study, is confirmed or whether, on the contrary, an equivalence of the two treatment methods can be assumed, independent of the underlying definition of abstinence and thus also with regard to PPA, as suggested by the results of the study by Carmody et al. (2008). In addition, it would also be worth exploring whether it would be possible to supplement hypnotherapy with relapse prevention strategies and achieve better outcomes.

The long-term abstinence rates achieved are similar to those also reported in previous controlled trials on the effectiveness of behavioral cessation programs (e.g., Hernandez-Lopez et al., 2009; Wittchen et al., 2011). However, these rates are lower than those achieved with a combination of behavioral therapy support and medication aids (Batra et al., 2008, 2010; Fiore et al., 2008). For example, in Batra et al. (2010), smoking abstinence was more than 30%, whereas both CA and 7 days PPA in this study was below 20%. While a number of studies have demonstrated the benefits of the combined use of behavioral therapy and pharmacological cessation aids (see Fiore et al., 2008; Stead et al., 2016), the combination of hypnotherapeutic cessation strategies with pharmacological aids has only been used in a small number of studies to date (Carmody et al., 2008; Schweizer and Revenstorf, 2008; Hasan et al., 2014). The question of whether a combination of hypnotherapeutic and pharmacological treatment methods can increase abstinence rates was only posed in Hasan et al. (2014) where hypnotherapy was compared to a nicotine replacement treatment, whereas the results of the study by Schweizer and Revenstorf (2008) provide evidence to the contrary. Future research is needed to determine whether hypnotherapeutic cessation concepts are as effective as cognitive-behavioral cessation concepts when combined with medications, and whether the addition of pharmacological support can increase the achieved abstinence rates to the same extent.

Another possible reason for the lower abstinence rates compared to previous studies of our research group may be lower treatment compliance. In the present study, only 54.6% of the study participants were highly compliant, whereas in the previous studies this had been the case for 81.0 and 73.5% of the study participants, respectively (see Batra et al., 2008, 2010). The comparatively lower treatment compliance may be related to the fact that two different treatment methods were compared, with more than 70% of participants preferring hypnotherapy. Many study participants may have hoped to be assigned to HT and might have been disappointed when randomization required them to undergo CBT treatment and vice versa. Perhaps the term “hypnosis” created a rather passive expectation of salvation. However, the two treatment conditions were not statistically different in terms of treatment use and treatment compliance, despite being preferred by a large proportion of the study participants and their reported higher efficacy ratings. We can only speculate that this may be due to the reality of HT treatment falling short of their expectation. However, compliance was a significant outcome predictor in our study; a higher number of sessions attended was consistently associated with a higher chance for abstinence from smoking. Both programs might therefor need to include more strategies to increase treatment adherence.

Analyses examining the influence of therapy-specific treatment expectancies on treatment outcome showed that a higher anticipation of the effectiveness of CBT had a positive effect on the probability of abstinence, whereas of the opposite was true for HT. This result was found regardless of the definition of abstinence used (CA versus PPA). This could be explained by a possible overestimation of the effectiveness of HT. In fact, participants rated the efficacy of HT significantly higher, which is contrary to current evidence (see Barnes et al., 2010, 2019). In addition, it has been reported that providers of hypnotherapeutic tobacco cessation treatments sometimes overstate the success rates of their cessation services (see Lynn et al., 2010; Yager, 2010), which may contribute to a potentially inflated assessment of the effectiveness of HT treatment by interested smoking cessation clients.

We found significant group differences in the 7 days PPA only when controlling for hypnotic suggestibility. Since no main effect for hypnotic suggestibility was found and there were no differences in suggestibility between HT and CBT at baseline, there might have been other factors such as practicing self-hypnosis at home in participants of HT that influenced this result. Homework, though, was not tracked in our treatments. In comparison with previous studies on hypnosis, Milling et al. (2007) found that suggestibility was a moderator for treatment outcome in patients with pain. Summarizing several studies on headache, panic disorder, and other clinical conditions in adults and children (Montgomery et al., 2011), suggestibility had a small to medium effect on outcome in HT. Focusing on smoking cessation, Lynn et al. (2003) reported that results on the influence of hypnotic suggestibility on treatment outcome are mixed.

### 4.1 Limitations

First of all, results cannot be generalized to Germany or Europe since data were obtained only in two study centers in Germany. Second, the number of therapists in the study centers was different, and in both centers, there was only one therapist offering HT treatment whereas there were more than two in CBT. Due to the complexity of the GEE model that already included the cluster structure of participants, we decided not to run additional analyses nested for therapists, and therefore do not know the (statistical) influence of therapists on outcome. Third, the fact that the study was advertised as a smoking cessation study with hypnotherapy may have played a role in the selection of subjects. As outlined in the introduction, interest in alternative treatments, especially hypnotherapy, is high among smokers (Sood et al., 2006; Tahiri et al., 2012) and may influence their treatment or outcome expectancies. Fourth, due to a reorganization of the study team, we were unable to publish results of the study earlier. Even if results were communicated to Barnes and used in their COCHRANE review (Barnes et al., 2019), the study data have now more than 10 years of age. Nevertheless, for two reasons, we are convinced that the study continues to be of great value and importance to the research community. The RCT was designed and conducted at a very high methodological level for the time, and, there are still far too few studies in the field of hypnotherapy in smoking cessation.

## 5 Conclusion

The present study provides evidence that the two cessation methods do not differ in their efficacy for long-term continuous abstinence from tobacco. The results of the present study may suggest that CBT treatment may be superior to HT treatment in terms of 7 days point-prevalence abstinence over the course of 12 months when taking into account hypnotic suggestibility, which appears to be clinically relevant. Future studies need to investigate whether the reported results of can be replicated. More than that, future studies should investigate for whom which treatment is most appropriate. The present study, thus, provides much needed robust data to evaluate the efficacy of a hypnotherapeutic tobacco cessation treatment compared to an established procedure. We conclude that HT – which is not current recommended as a first line intervention for the treatment of tobacco dependence – can be an effective alternative treatment option when CBT or other conventional treatments are being refused. It may well be that very different target groups of smokers are reached and therefore hypnotherapy as a therapeutic method is an important addition to the existing and established procedures of smoking cessation. As meta-analyses have shown, HT might have additional effects on the efficacy of CBT and, thus, can also be combined (e.g., Ramondo et al., 2021). Future studies should assess, if abstinence rates in both treatments could be enhanced with a shared decision-making approach following patients’ preferences. The results of the current study provide an important argument for providers of hypnotherapeutic tobacco cessation services and evidence for future revisions of recommendations in national and international treatment guidelines. Based on reliable data, it can now be stated that hypnotherapeutic methods for smoking cessation can, under certain conditions, be comparable in effectiveness to established methods such as CBT.

## Data availability statement

The raw data supporting the conclusions of this article will be made available by the authors, without undue reservation.

## Ethics statement

The studies involving humans were approved by Ethics Committee for Behavioral Research of the Medical Faculty of the Eberhard Karls University of Tuebingen (331/2008B01) and the Ethics Committee of the Medical Association of Hamburg (MC-150/10). The studies were conducted in accordance with the local legislation and institutional requirements. The participants provided their written informed consent to participate in this study.

## Author contributions

AB: Conceptualization, Funding acquisition, Methodology, Project administration, Resources, Supervision, Writing – original draft, Writing – review & editing. SE: Investigation, Methodology, Project administration, Formal analysis, Visualization, Writing – original draft, Writing – review & editing. BR: Investigation, Methodology, Writing – review & editing. SF: Investigation, Writing – review & editing. KF: Formal analysis, Writing – original draft. IT: Conceptualization, Writing – review & editing. ST: Conceptualization, Methodology, Supervision, Writing – review & editing.
