# Supplementary material for: Hypnotherapy compared to cognitive-behavioral therapy for smoking cessation in a randomized controlled trial
Source: Front Psychol. 2024 Feb 27;15:1330362. doi: 10.3389/fpsyg.2024.1330362 (PMC10929270; doi:10.3389/fpsyg.2024.1330362)
Supplement: Supplementary file 1 [file Table_1.DOCX]

Supplementary Material. *CBT and HT Treatment Session Timeline.*

|  | *CBT Treatment Session Timeline* | | HT Treatment Session Timeline. | |
| --- | --- | --- | --- | --- |
| Session | Main treatment/study components | | Main treatment/study components | |
| Week 0 | - Information regarding study content and timeline - Informed consent - Questionnaires | | - Information regarding study content and timeline - Informed consent - Questionnaires | |
| Week 1 | - Greeting - Questionnaires - Introduction of CO test - CO test* - Treatment timeline, organization - Today’s agenda* - Introductions of Participants (smoking history, motivation, typical daily smoking situations) | - Group decisional balance exercise - Psychoeducation: Nicotine/tobacco dependence - Explanation of homework* (1^st^ week: self-monitoring) - Preview of next week’s session* - Feedback round* | - Greeting - Questionnaires - Introduction of CO test - CO test* - Treatment timeline, organization - Today’s agenda* - Introductions of Participants (smoking history, motivation, typical daily smoking situations) | - Introduction: What is hypnosis? - Group task: Searching for attractive terms to describe the desired state. - Trance: Creating a future vision of oneself as an abstinent person - Preview of next week’s session* - Feedback round* |
| Week 2 | - Group check-in* - Typical smoking situations and alternatives to smoking - Introduction of the motivational statement | - Behavioral tips for the 1^st^ smoke-free day - Set date for 1^st^ smoke-free day | - Group check-in* - Self-reports (trance experiences in week 1, smoking behavior since week 1) - Explanation: Day X as starting date of a smokefree future and strategies to reach it | - Trance: Imagining the positive effects of nonsmoking, imagining date of Day X, ideodynamic signals - Confirming each individual date during the next week in the group - Homework: Practicing the imagination of a smokefree future |
| Week 3 | - Use of rewards/pleasurable activities - Course “buddy”/setting up behavioral contracts and bets | - Healthy eating/lifestyle | - Self-reports (Day X, first days without cigarettes, relapses) - Trance: Ideomotor action to work on ambivalence and stop increasing weight | - Alternative rituals - Homework: Imagination exercise to work against increasing weight |
| Week 4 | - Feedback on positive changes since smoke-free (motivational enhancement) | - Review smoking alternatives and how these have worked (support self-efficacy) - Risky situations/solutions | - Trance: Identifying impulses for and against smoking, growing distance to smoking behavior | - Trance: Self-hypnosis training (future imagination and connecting the desired outcome to potential relapse situations) - Homework: Self-hypnosis training |
| Week 5 | - Review of key intervention elements - Relaxation training |  | - Trance: Individual Self-hypnosis training with each group member |  |
| Week 6 | - Review of key intervention elements - Relapse dynamics: slip, lapse, relapse | - Relapse prevention - Crisis plan in case of relapse - Feedback round/Wrap-up | - Trance: Ideodynamic farewell, imagination of a free identity | - Feedback round/Wrap-up |

Notes. * = indicates an element repeated in all subsequent weeks.
